# Facts vs. Theories

**Published:** 1870-03

**Authors:** Jas. Taylor

**Affiliations:** President of the Board of Trustees


DENTAL REGISTER.

VOL. XXIV.] MARCH, 1870. [NO. 3.

### Original Communications.

### FACTS *vs.* THEORIES.

ADDRESS TO THE GRADUATING CLASS OF THE OHIO COLLEGE OF DENTAL SURGERY, BY DR. JAS. TAYLOR, PRESIDENT OF THE BOARD OF TRUSTEES, MARCH 3rd, 1870.

Facts are stubborn things. They are the immutable truths on which true science is anchored—the imperishable—sifted from the superstition and dross of ages. The gleaning process still goes on; and within the last quarter of a century an army of Dental explorers have entered the field, and with zeal, energy and great perseverance, are hewing out solid blocks of granite *facts* as foundation-stones for a noble science.

We are glad to see recruits for this army, and hail with pleasure every graduating class as they pass from these halls and take their place with the veterans of the service.

Facts, gentlemen, are not only stubborn things, but they are very useful; they will be excellent guides in your practice; if neglected and trampled under foot, they will rise up and at some turn or corner stare you in the face.

They are not only useful, but when polished by friction with error they sparkle as gems of the first water. They are the diamonds of our science, of far more value than all the gems of the most costly crown.

What, then, are the facts of Dental science?

First, then, it is an established fact that the teeth are regularly organized bodies, dependent on the general system for vitality, and subject to the same laws which govern the general system in growth and elaboration. This would appear to be a self-evident truth; yet the theory was once entertained that they were a kind of extraneous bodies—the permanent ones developed from the roots of the deciduous organs.

This foundation principle gives us Dental surgery. Every operation on the teeth is a surgical operation.

The second fact runs back to a time of which we know not, when these organs partook of the virus of sin and first showed evidence of disease.

The third fact is, that Dental caries is a destructive disease, involving the loss of these organs, as well as much general constitutional disturbance. This disease made necessary a class of Dental operators, whose practice was in singular opposition to the theories they entertained. Their practice was far in advance of their theories. Their practice was predicated on the result of experience, which, in its unvarying results, accumulated evidence which helped to disprove all the speculations of false theory.

We allude to the theory of Hunter and Fox, that caries was the result of inflammation of Dental structure. This being true, filling the cavity even after thorough extirpation of the carious portion, could not arrest the further progress of the disease, for the primary cause would still exist. There is nothing in gold, tin, or even lead, as then often used, which could exert a restraining influence on the disease.

Fact fourth is, that dental caries is local, external and chemical, and that imperfect dental development renders these organs more susceptible to this influence. It is also a demonstrated fact that the teeth, when bathed only by normal secretions, can be preserved. That gold is the best material yet found for filling teeth, and that amalgam is the poorest stuff ever yet introduced, and also that the operator who gives himself up to the use of this vile compound for filling teeth will never attain excellence in the use of gold. It is a Dental incompatibility.

It is a fact that excellent Dental operations are far more frequent now than thirty years ago. Yet it is indisputable that Hudson, Gardett, Keoker, Hayden, Putnam, Harris, Parmly and a few other such, have done more to consolidate and establish the importance and utility of our profession than one surrounded with the advantages which you gentlemen now possess can well imagine.

We must recollect that the most of these, indeed, all, spent the prime of their professional life without a Dental literature. They commenced their labors amidst the most profound ignorance and superstition, when nostrums, charms and incantations were more frequently the cure for odontalgia than anything else; when three-fourths of the public regarded Dentistry as a mere ornamental art. Even medical science, with all its accumulated lore of ages, had spent scarce a thought on curative De tistry. True, they often found the teeth troublesome organs to manage, and equally troublesome to extract. It is a singular fact that no other part of the system was so neglected. This is the more strange when anatomically and physiologically some master minds had thrown the light of research upon them. Still, practically, no special attention had been bestowed by the profession on the means for their preservation. Prejudice had consigned these gems to the unmerciful slaughter-house of the barber.

We can imagine a celebrated surgeon in the Imperial City opening an abscess, or delicately removing a needle or splinter from the toe of a patient; and then to save his dignity sending him to a barber to have a tooth extracted. Hunter,

Fox and Bichat were about the first to expose this folly and awaken some attention to this important branch of the healing art.

It was well for the profession that at such a time such men as we have named entered the profession, and that they were in this New World, free from the prejudices of the Old. We can not look back on those master minds without admiration ; and when we note their indefatigable perseverance, their diligent search for truth, their careful observation of the results of practice, their practical good sense in following the lessons of experience, their comprehensive views of general science as contributary to Dental science, their years of labor and toil in laying broad and deep the foundations of a noble profession, their high-toned, self-conscious pride of character commanding respect among all classes, and to all this that excellence of work which long outlasts their own lives, and will remain as long as their patients shall live as monuments of their professional skill, we stand in awe and reverence, bow and do homage to their worth.

We admit that, in the operation of filling teeth, we now tax our science with a greater work, and claim to, and do accomplish, more than those then undertook.

Still, let us not exult over our success until thirty years of wearing and grinding shall tell their tale upon our work. We like to see a fifty-year-old clock which has passed carefully through the manipulation of a faithful workman, standing in its big upright case in the old family corner—its long pendulum swaying backward and forward, measuring out time by the second; and with its continued tick, tick, tick—reminding youth that time is passing, and old age that the years are moving onward to the grave.

The tooth of time has made but little wear on the faithful chronicler, and we can not but hope it may still measure out the life of many generations.

So when we see a golden gem packed carefully away fifty years ago, by one of these masters, we can not but wish the owner might live for ages to see how long this trophy of artistic skill might last.

We have more large, finely polished gold fillings now, than in the time of our fathers. We have also more miserable compound and amalgam fillings now than then. We have fewer faithful solid tin fillings now than then; and we venture to affirm that their cheap operations in this way were far better then than now. It is a matter of great doubt if we have any cheap material for filling teeth at all comparable to tin. It is a fact, however, gentlemen, that Dental science has made great progress in the last thirty years, and there never was a time when the profession was so well armed for future progress. We have now a literature of our own, and it is for you to make this a power for good or evil. Sustained aright with brains and money, it will be an instrument of great progress; used as a selfish means of personal aggrandizement, it will be a clog in the wheels of science.

There is scarce a branch of general science, mechanical as well as natural, but are contributing rich stores of knowledge of essential service to the Dentist.

It is a fact, that in the treatment of exposed nerves, we now attempt, and accomplish more, than formerly. Fang filling is far better understood now than then; yet fang drilling obliquely through to the walls of the alveolus is far more common now than then. Capping of exposed nerves was not, however, then unknown; and the use of the actual cautery was far better understood then than now. Extirpation of the nerves was practiced with great success, and required far more skill than is now necessary in removing a devitalized one. It was a heroic age and a heroic practice based on the general principles of surgical science. No letheon draught threw its potent spell over the patient, and no polished forceps had yet dethroned the turnkey. Gentlemen, from that day to this, the best method of treating exposed nerves has been, and is still, a mooted question.

Theory, speculation and experiment have not, as yet, determined all the facts of the case, and we wait the results of practice. No practice as yet adopted can claim universal success. There are certain principles of physiology and pathology which should convince every reflecting mind that no treatment can in all cases positively insure this.

It would be an interesting, and perhaps profitable, work to commence with the first number of the *American Journal of Dental Science* and read all which this and other periodicals have published on this subject.

The searing of the exposed surface of the pulps was at one time recommended and practiced, the object being to merely obtund the exposed surface so that the filling could be introduced and the nerve not destroyed. But these seared nerves would inflame and die; facts were stronger than theories.

Next, a fine gold cap was arched over the nerve. It was thought a golden crown would not only shield the organ from the presence of the plug, but please and quiet the captious complaining member, and leave it free to perform its duty without molestation. Still it would not always be thus satisfied. It did not like confined air or so much room to play in. Then it was thought a fine, delicate piece of oiled silk would better please this whimsical organ; but it was no more acceptable than gold. Bone, rubber, and almost everything which could be thought of has been applied, and still this little refractory nerve would not be satisfied, and keep quiet. At length, tired and vexed with this continued wooing and coaxing, a bold and determined operator determined to try the effect of the lash, and see if he could not whip it into subjection. So he took a delicate drill and started it under the edge of the gum and drilled into the dental foramen, cutting the nerve. This was a new mode of attack, and caused a kind of surrender. Some thought that it shrunk up in the crown of the tooth, and retired into the root to recuperate, and then would ever after behave itself. Others thought it would re-unite and soon be as lively and cheerful as ever, only not give any more trouble; but stubborn fact soon settled risodontrophy, and, in justice to Dr. Hullihen, we would say that he never appeared to be half as sanguine of its success as many others. Risodontrophy failing, and capping uncertain, the sentence appeared to be death and extirpation. Theory adapting itself to the necessities of the case, argued that the nerve was not essential to the integrity of the tooth, for the periosteum supplied nourishment to the cementum, and the vital union between that and the dentine was of so low a character that no positive injury could result by cutting off all vital connection between them. Dead teeth were not foreign bodies, or the old notion that living healthy organism will repel such structure must be false. We know that the mortified part must be sloughed off before the parts can be restored.

Claiming dental caries to be essentially different from mortification, it would appear that infinite wisdom had so constructed the dental organs as to meet the fact that dental caries shall exist, and even partial death take place without their utter loss.

Gentlemen, we allude to this subject to show that there is much theory and speculation to be settled before practice may be considered definitely fixed on this subject. We are now just in the midst of experiments, trying the wonderful charms of oxychloride of zinc, which, in the fond imagination of some, is to assimilate itself to the pulp tissue so admirably as to completely revolutionize our treatment of nerves, and consign to the tomb all past barbarous practice. It is our mission to cure, not to kill; to save, and not destroy. This is now the great nerve tranquilizer, restoring lost tissue, moulding itself to this delicate organ, soothing all irritation, and making the lame well again.

But, gentlemen, theories are not facts; and you will find that unless your theories are sustained by facts they will soon crumble beneath the touch of time, and all your fond imaginings be in wrecks about you.

Theory sometimes helps to develop facts. Yet how often is fact thus obscured and covered up so thick under the rubbish of speculation that years of assiduous toil and labor is necessary to bring to light the simple truth.

There is always a cause antecedent to an effect; yet the effect seen does not by any means always reveal the cause. Caries is a tangible effect of some cause ; yet centuries likely passed before the cause was truly found out.

Theory here, in all probability, retarded the discovery of the cause, and may have prevented, for ages, the proper remedy.

Theories excite much discussion, but facts stop all argument. Facts are to be treasured, and, when once revealed, should be carefully guarded, and never again hid by the sophistries of speculation.

We have thus merely glanced at the present status of operative Dentistry. The progress made gives good cause for your encouragement, and also holds out inducements for further effort. There is much to stimulate research; much to reward diligent effort and excite professional emulation.

The facts of surgical Dentistry make it more and more apparent every year that it is a very important branch of the healing art, and that it becomes more and more identified with general surgery; while mechanical Dentistry has of late taken the other track and affiliates with mechanic art.

We can, with pride, refer to a period when mechanical Dentistry received more attention, and made more rapid advancement than any other department of the profession; when progress was the order of the day; when chemistry and metallurgy were indispensable auxiliaries; when durability and indestructibility were written of, spoken of and aimed at, when the great effort of art was to rival nature; when a dental laboratory was a museum of curiosity; when true science and art were so blended that the most celebrated painter's studio hardly rivaled the artistic taste and skill displayed. Did the painter study color and shade? so did the Dentist; did he study form and ease of position? so did the Dentist; did he aim at grace and expression? so did the Dentist; one was an art sketching on canvass, the *fac simile* of nature; the other, an art grafting on to nature a *fac simile* of her own work.

The best material and the best work was the desideratum to be obtained, and we venture to affirm that no twenty years of our professional history will ever show a greater work, and more progress, than that immediately succeeding the introduction of porcelain teeth.

What, let me ask, is mechanical Dentistry to-day? Look at the stereotyped edition of her work all around us! We might say, with propriety, that God never exactly stereotypes the human countenance. Look at the millions of our race, and see variety everywhere. Variety and harmony is the law of nature; how beautifully shade and color blend, and nature's pencil ever striving to hide deformity.

Mechanical Dentistry is sadly getting out of joint. Five or six editions of teeth—we believe the last not yet out—stereotypes the rubber catalogue, and these for the million; and if block teeth and rubber plate hold out ten years longer, if people do not look very much alike it will not be the fault of our mechanics.

We ventured the assertion, near twenty years since, that mechanical geniuses were the curse of the profession. We thought we saw then the tendency, in a great many, to rely upon their special mechanical skill for success. They could not but succeed, for they were born operators. Nature had turned them out almost ready for work. There was no need of learning what they already knew. Their superior *tact* was above all knowledge of others.

Our fear was that nature's work might never be improved, and thus true science would be utterly neglected. Is not mechanical Dentistry at this time passing through this ordeal ?

Had one of our old practitioners concluded, twenty years ago, to take a Rip Van Winkle sleep in his laboratory, and should some modern operator, during his slumber, remove the fine appliances of his skill, introducing his engine-vulcanizer, &c , &c., and now awake him, amidst the fumes of sulphur, with the raspers and scrapers at work, we doubt not his situation would be full, if not more, embarrassing than that of the celebrated sleeper.

If rubber, and sulphur and amalgam represent the lowest depths of our profession, gold and platinum may, with equal propriety, represent the highest excellence.

It may be asked, what does all this amount to ? We answer, that rubber, like amalgam, is a cheap and vicious article, and neither can take the place of gold in general practice.

In conclusion, allow me to refer to a few facts especially applicable to success in your chosen profession.

Your duties are of a delicate nature, very different from the rough pursuits of ordinary life. We hold that every man should array and keep himself in accordance with the duties of his calling. There are some avocations where clean hands and broad cloth would be out of place, and the nature of which demand that which is rough and strong, and willing hands to accomplish the labor.

We are willing to come in contact with clean hands and gentle treatment. The bloody instrument of yesterday is disgusting and horrible for use to-day without thorough cleaning.

The fumes of tobacco may be pleasant to the one who makes them, but to many they are perfectly sickening. lager beer, wine and brandy are poor aids to the Dentist, and he who sees fit to wallow in these *delicacies*, and soak and smoke himself in such luxuries, must expect to be packed away for use in some other way. The fact is, the practice of Dentistry and these things are incompatible.

In connection with these thoughts, there are a hundred little things which suggest themselves—such as clean hands, clean instruments, clean napkins, clean persons, pure water, pure speech, pleasant address, cultivated taste, refined manner and *gentle firmness*, which all go to smooth and render more easy the professional career of any practitioner.

The discussion of these alone would take more time than is appropriate for an address of this kind, and hence we merely suggest them for your future consideration.

You commence practice, gentlemen, with the highest endorsement of this institution; and yet this diploma confers no merit. You might possess all the knowledge of all these professors, and yet waste it to no purpose; but, possessing all this, with their merit and due regard to all these little things, and your success is certain.

